# Contrasting in vitro vs. in vivo effects of a cell membrane-specific CC-chemokine binding protein on macrophage chemotaxis

**DOI:** 10.1007/s00109-014-1194-6

**Published:** 2014-08-01

**Authors:** Eileen McNeill, Asif J. Iqbal, Jyoti Patel, Gemma E. White, Daniel Regan-Komito, David R. Greaves, Keith M. Channon

**Affiliations:** 1 Division of Cardiovascular Medicine, John Radcliffe Hospital, University of Oxford, Oxford, OX3 9DU UK; 2 Wellcome Trust Centre for Human Genetics, University of Oxford, Oxford, UK; 3 Sir William Dunn School of Pathology, University of Oxford, Oxford, UK

**Keywords:** Chemokine, Macrophage, Therapy, Inflammation, Transgenic

## Abstract

**Abstract:**

Chemokines (CK) provide directional cues that mediate the recruitment of leukocytes to sites of inflammation. Broad-spectrum blockade of the CC-CK family, using the vaccinia virus 35K protein, has been shown to cause a potent reduction of systemic inflammation in models of atherosclerosis, vein graft disease and arthritis. We have used a cell membrane-targeted form of 35K, Mem35K, to probe whether cell-associated blockade of chemokine response is sufficient to reduce cell recruitment in inflammation. In Tie2cre mice, activation of a flox-stop Mem35K transgene directed conditional expression of Mem35K in leukocytes and endothelial cells, confirmed by Western blotting, flow cytometry and immunofluorescence microscopy. This conditional Mem35K expression was sufficient to increase cell surface CCL5 binding and reduce chemotaxis in vitro to CCL5, CCL2 and CCL3 but not to non-CC-CK chemoattractants, LTB4, C5a or chemerin. However, in vivo monocyte recruitment into the peritoneum driven by zymosan or CC-chemokine injection, which was demonstrated to be CC-CK dependent using CCR2−/− mice, was not reduced by Mem35K expression, despite the expression of functional Mem35K protein. These findings highlight differing requirements for cell-associated anti-inflammatory activity in in vitro and in vivo models.

**Key message:**

- Mem35K is a cell-associated CC-chemokine binding protein.
- Conditional Mem35K transgenic mice show expression Mem35K in leukocytes.
- Mem35K blocks in vitro primary macrophage chemotaxis specifically towards CC-chemokines.
- Mem35K expression is not sufficient to reduce inflammation in vivo.
- The requirements for anti-inflammatory activity in vitro and in vivo are different.

**Electronic supplementary material:**

The online version of this article (doi:10.1007/s00109-014-1194-6) contains supplementary material, which is available to authorized users.

## Introduction

CC-Chemokines (CC-CK) are a large class of low molecular weight 8–12 kDa chemoattractant cytokines that predominantly mediate the migration of mononuclear cells [1]. CC-CK ligate GPCR receptors, activating downstream signalling and leading to cellular activation, cytoskeletal reorganisation and migration [2]. Studies in mice deficient in CC-CK or their receptors have demonstrated a pivotal role for CC-CK signalling in mediating cell recruitment in multiple immune and inflammatory models including atherosclerosis, experimental autoimmune encephalomyelitis (EAE), asthma and graft versus host disease [3–6].

Viruses have evolved a portfolio of immune defence molecules that can block or modulate the mammalian chemokine system. Pox family viruses express a soluble 35-kDa protein, ‘35K’, that binds and inactivates almost all members of the CC-chemokine family [7]. Analysis of the purified protein shows it binds all human and mouse CC-CK with high affinity [8]. The crystal structure of 35K bound to CCL4 reveals that when the chemokine is bound, the residues required for receptor binding are obscured [8]. Systemic treatment of animals with 35K via hepatic gene transfer or with a soluble Fc-fusion protein form of this molecule is able to reduce disease in models of atherosclerosis, fibrosis, arthritis and allergic airway inflammation [7, 9–11]. The potency of this chemokine-binding molecule makes it an attractive tool for determining the role of CC-CK in disease models and for probing the therapeutic potential of broad-spectrum CC-CK blockade in experimental disease models. In cardiovascular disease in particular, there is evidence that multiple CC-CK and their receptors act in concert to promote atherosclerosis, since CCR2^−/−^, CCR5^−/−^ and CCL2^−/−^ mice have all shown reduced atherosclerosis in hyperlipidemic models [4, 12, 13]. Indeed, the combined inhibition of both CCL2 and CCL5 activity led to additive inhibition of atherosclerosis [13]. It is not yet clear whether the potency of multiple CC-CK blockade is indicative of functional redundancy within the CC-CK system or is a result of a complex pathology manifesting through multiple parallel highly cell-specific roles for individual CC-CKs [14]. Indeed, small-molecule CC-CK inhibitors are already being tested in clinical trials as therapies for inflammatory diseases, but there is uncertainty as to whether local, cell-specific or systemic inhibition of CC-CK action will be necessary for clinical efficacy, whilst avoiding potentially harmful effects on systemic immune function. Thus, there remains a pressing need to understand how cell-specific effects of CC-CK inhibition will impact on inflammatory cell recruitment.

To test the potential of CC-CK inhibition as an anti-inflammatory strategy targeting individual cells, we have engineered a novel, cell-localised form of the 35K chemokine binding protein, Mem35K, that has a cell-restricted expression pattern through a FasL transmembrane domain, facilitating localization to membranes, and a green fluorescent protein (GFP) moiety for cellular identification [15]. Cell transfection studies and adenoviral delivery to the liver demonstrated that high-level expression of this molecule is sufficient to reduce the chemotaxis of Mem35k-expressing cells and can reduce hepatic inflammation in the concavalin A model of hepatitis [15]. However, adenoviral delivery of a cell-localised molecule is a fundamentally limited strategy, since the principal target of gene transfer techniques such as adenoviral delivery is the liver. Whilst this is ideal for the production of secreted molecules, or effects mediated by high-level hepatocyte transgene expression, it does not allow the targeting of specific cell types such as leukocytes. Furthermore, the important question of whether CC-CK inhibition can be achieved at the level of individual inflammatory cell populations, as distinct from whole-organ or systemic effects, remains unknown.

To address these questions, we now report the development of a system for cell- or tissue-restricted expression of Mem35K, using a conditional transgenic mouse for expression of Mem35K under control of a CMV flox-stop promoter. To evaluate the transgenic cell-localised expression of Mem35K, we crossed these mice with transgenic mice expressing cre under control of the Tie2 promoter, in order to direct transgenic Mem35 expression to leukocytes.

## Materials and methods

Additional methods can be found in the Supplementary Material.

### Generation of the Mem35K transgenic mouse

The Mem35K mouse was commercially produced by genOway (Lyon, France). The Mem35K cDNA was introduced into the *Hprt* locus using the ‘Quick Knock-in’ targeting vector containing the CCAG promoter and a validated floxed STOP cassette [16] and the human HPRT allele to reconstitute the *Hprt* gene. The targeting cassette was linearised, isolated and purified prior to electroporation into E14Tg2a ES cells derived from 129P2/Ola mice. Positive selection was achieved by identification of HAT-resistant clones. Southern blotting identified 9 ES cell clones that were correctly targeted. The recombined ES cells were injected into blastocysts from pseudopregnant C57bl/6J mice. Chimeric male offspring with 80–100 % chimerism were selected for breeding to confirm germline transmission. Two founder 80 % chimeric males demonstrated germline transmission and produced 8 female Mem35K heterozygous mice.

## Results

### Mem35K elicits GFP fluorescence, membrane-localised 35K protein and reduces CC-chemokine receptor-mediated chemotaxis

**Fig. 1:**
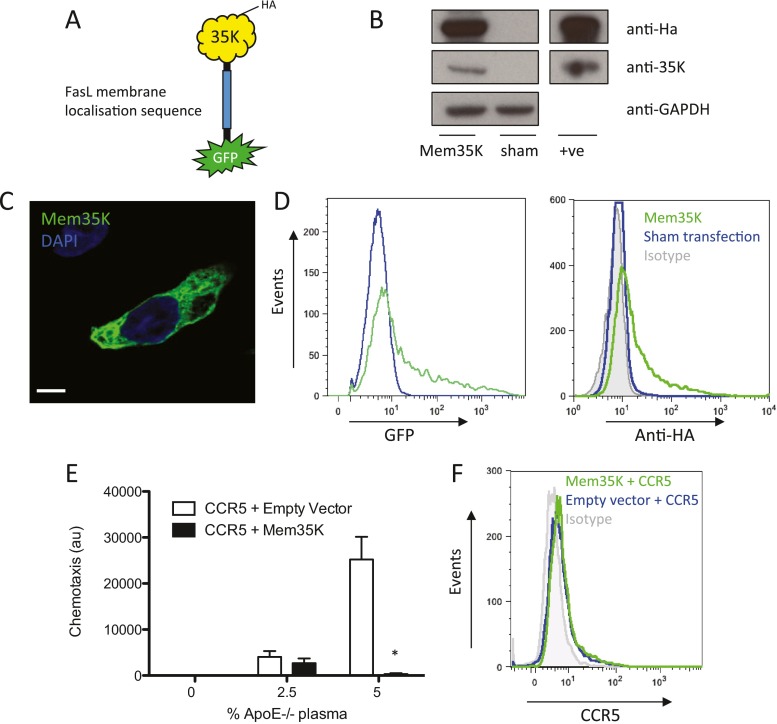
Confirmation of the Mem35K protein structure. **a** Schematic representation of the Mem35K molecule. **b** Expression of a Mem35K plasmid in HEK293 cells resulted in expression of a 65-kDa protein that was detectable by Western blotting with an anti-HA or anti-35K antibody and absent in sham-transfected cell lysates (loading control GAPDH). Positive controls of a cell line stably expressing the GTCPH-HA molecule and recombinant 35K-Fc protein were used to confirm antibody specificity. **c** Transfected cells showed endogenous GFP fluorescence with a membrane-associated expression pattern. (scale bar 2 um). **d** To confirm cell surface expression of a proportion of the Mem35K molecule, transfected HEK293 cells underwent cell surface staining with anti-HA antibody or isotype control (*grey histogram*). Endogenous GFP fluorescence was also quantified to confirm transfection. **e** Functional Mem35K expression was confirmed by coexpression in a CCR5 bioassay, with migration of CCR5 expressing HEK 293 cells towards ApoE^−/−^ plasma being inhibited by coexpression of the Mem35K molecule. (*p < 0.05, cells assayed in duplicate towards *n* = 3 independent plasma samples per experiment and replicated in two independent experiments). **f** Cells co-transfected with Mem35K or empty vector and CCR5 expressed similar levels of CCR5 on the cell surface, as detected by flow cytometry using an anti-CCR5 antibody

In order to validate the functional effects of the transgenic Mem35K protein, HEK 293 cells were transfected with a plasmid encoding Mem35K, incorporating intracellular N terminal GFP and FasL transmembrane domains, fused with extracellular 35K and C terminal HA epitope tag (Fig. 1a). Western blotting of cells 24 h after transfection demonstrated the presence of the expected 65-kDa Mem35K protein, which was detected with antibodies targeted against either the HA epitope tag or the 35K molecule (Fig. 1b). To confirm the presence of GFP within the construct, fluorescence microscopy and flow cytometry were used to detect GFP (Fig. 1c, d). The cell membrane localisation and functional expression of Mem35K were confirmed by confocal microscopy to visualise the intracellular GFP, which showed a non-ubiquitous localised distribution (Fig. 1c) within cell membranes through the cell. To confirm the presence of mem35K expression on the cell surface membrane, which is required for activity, we performed flow cytometry with an anti-HA antibody and demonstrated cell surface HA in the Mem35K-transfected cells (Fig. 1d). To test the effects of Mem35K molecule on chemotaxis towards biologically relevant stimuli, we compared the chemotaxis of HEK 293 cells, transfected with either CCR5 alone or co-transfected with CCR5 and Mem35K, in response to plasma from ApoE^−/−^ mice, which has high plasma CC-CK activity. CCR5-transfected HEK 293 cells showed significant migration towards ApoE^−/−^ plasma, at either 2.5 or 5 % in chemotaxis buffer (Fig. 1e). This migration of CCR5-expressing cells was significantly inhibited by cotransfection with Mem35K (*p* < 0.05). The striking inhibition of chemotaxis by Mem35K transfection, despite typical transfections efficiencies of >50 %, is likely to indicate an inhibitory effect on both the expressing cells and exertion of a bystander effect on non-expressing cells, as we have documented previously [15]. Flow cytometry using an anti-CCR5 antibody confirmed CCR5 expression was unchanged between groups (Fig. 1f).

### Transgenic expression of Mem35K

**Fig. 2:**
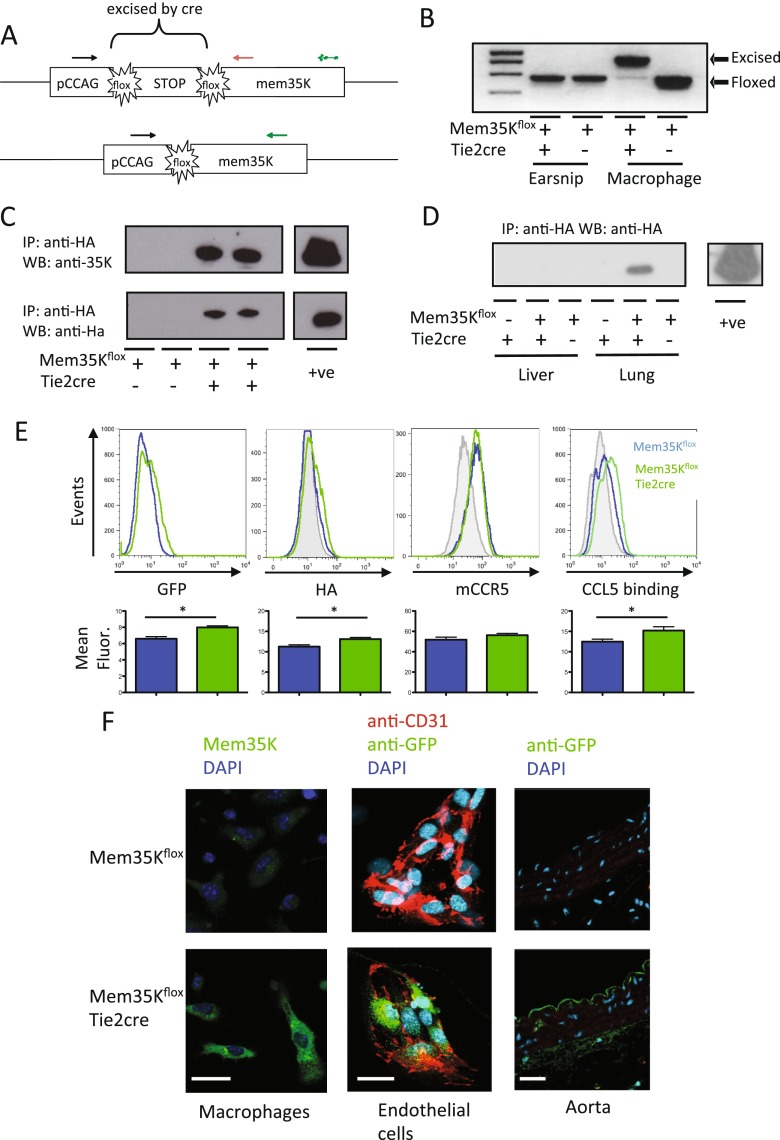
Production of a Mem35K expression transgenic mouse. **a** Schematic representation of the Mem35K-floxed allele in both the floxed and cre-excised form. Genotyping primers are shown: shared primer as *black arrows*, the floxed allele is formed by primers marked as the *black* and *red arrows* and the excised allele by the *black* and *green arrows* with the *dotted green arrow* representing a longer PCR product that is not produced by the *black* and *green primers* in the absence of cre-mediated excision of the floxed DNA segment. **b** PCR analysis of genomic DNA extracted from Mem35K-floxed animals in the presence or absence of the Tie2cre transgene showed the excised ‘active’ allele is not detected in ear snip DNA, but it is efficiently detected in primary macrophages. **c** Immunoprecipitation using anti-HA agarose beads followed by Western blotting identified a protein product only in Biogel-elicited macrophages from animals carrying both the Mem35K^flox^ and Tie2cre transgenes, identified using both an anti-35K and anti-HA antibody. Positive controls GTPCH-Ha (anti-HA) and recombinant 35K-Fc (anti-35K). **d** The Mem35K protein was detectable by anti-HA immunoprecipitation followed by Western blotting using an anti-HA antibody in protein lysates from endothelial and leukocyte-rich lung tissue and not from liver in animals carrying both Mem35K^flox^ and Tie2cre. **e** Primary Biogel-elicited macrophages from Mem35K^flox^ Tie2cre animals showed significantly higher GFP fluorescence than Mem35K^flox^ animals by flow cytometry (*n* = 6 per genotype, *p < 0.05) as well as significant expression of the HA epitope on the cell surface (*n* = 3 per genotype, *p < 0.05). The expression of the CCR5 was unchanged (*n* = 3), but the Mem35KfloxTie2cre animals demonstrated greater CCL5 binding to the cell surface (isotype or control protein binding shown in *grey*) (*n* = 6, *p < 0.05). **f** Immunofluorescence confocal microscopy demonstrated endogenous GFP fluorescence in Biogel-elicited macrophages that was not present in the absence of cre. Anti-GFP immunofluorescence demonstrated GFP expression in primary endothelial cells and aortic sections from Mem35K^flox^ Tie2cre animals. *Scale bar* 10 um

To generate a transgenic mouse, the Mem35K transgene was cloned into an expression cassette under control of the ubiquitous CCAG promoter (CMV immediate early enhancer/chicken β-actin promoter fusion). A *lox*P flanked STOP cassette was included between the promoter and the Mem35K transgene to allow conditional gene expression (Fig. 2a). The Mem35K transgene was targeted to the *Hprt* locus, on the X chromosome, by homologus recombination. To ascertain the integrity of the flox-stop system, Mem35K^flox^ mice were crossed with mice expressing cre under control of the Tie2 promoter (Tie2cre mice). These mice express cre in a shared haematopoietic/endothelial progenitor population, resulting in cre-mediated DNA deletion in all mature leukocytes arising from this population, as well as in endothelial cells [17, 18]. Tie2cre also demonstrates cre expression in the female germline; thus, only male Tie2cre animals are used for breeding to maintain conditional gene expression [18, 19]. Primers were designed to detect both the floxed and excised Mem35K alleles. In Mem35K^flox^ Tie2cre mice, conditional gene expression was demonstrated with earsnip DNA showing only the floxed product, but in macrophages there was efficient cre-mediated production of the excised allele (Fig. 2b). To confirm that the CCAG promoter was able to drive Mem35K protein production, immunoprecipitation was performed using anti-HA conjugated agarose beads in lysates produced from primary peritoneal macrophages elicited by Biogel from Mem35K^flox^ Tie2cre mice (Fig. 2c). No Mem35K protein was detected in the absence of cre expression, indicating that the STOP cassette efficiently prevented gene expression. In animals co-expressing cre, immunoprecipitated Mem35K protein was detected using either anti-HA or anti-35K antibodies in Western blotting. Furthermore, Mem35K protein was detected by immunoprecipitation in the lungs, where endothelial cells and leukocytes are abundant, but was below the limits of detection in organs such as the liver where other cell types predominate (Fig. 2d).

### Functional transgenic expression of Mem35K detected in vitro

To further investigate the expression and localization of Mem35K protein in cells from Mem35K^flox^ Tie2cre mice, we performed fluorescence confocal microscopy on primary macrophages ex vivo, which had demonstrated Mem35K protein expression by Western blotting. Endogenous GFP fluorescence was detected in Biogel-elicited peritoneal macrophages from mice expressing Mem35K, by both significantly increased green fluorescence by flow cytometry (Fig. 2e) and confocal microscopy (2F), although expression was not sufficiently high to show clear localization of the transgene within the cell. The localization of mem35K to the cell surface was confirmed by detection of the HA epitope on the cell surface by flow cytometry (2F). The presence of active mem35K on the cell surface was confirmed by detection of significantly increased biotinylated CCL5 binding to the cell surface (Fig. 2f). Mem35K^flox^ Tie2cre mice yielded macrophages with a greater binding capacity for CCL5. Furthermore, we confirmed that this effect was not due to alterations in cell surface chemokine receptor expression by detecting no significant difference in CCR5 expression on the cell surface using an anti-CCR5 antibody (Fig. 2f). In primary endothelial cells from Mem35K^flox^ Tie2cre mice, identified using anti-CD31 immunohistochemistry, expression of the Mem35K molecule was revealed by anti-GFP immunohistochemistry, but only in Mem35K^flox^ Tie2cre mice that carried both the Mem35K and Tie2cre transgenes (Fig. 2f). Endothelial expression of Mem35K was also demonstrated using anti-GFP immunohistochemistry in the aorta from Mem35K^flox^ Tie2cre mice, compared to non-expressing controls (Fig. 2f).

**Fig. 3:**
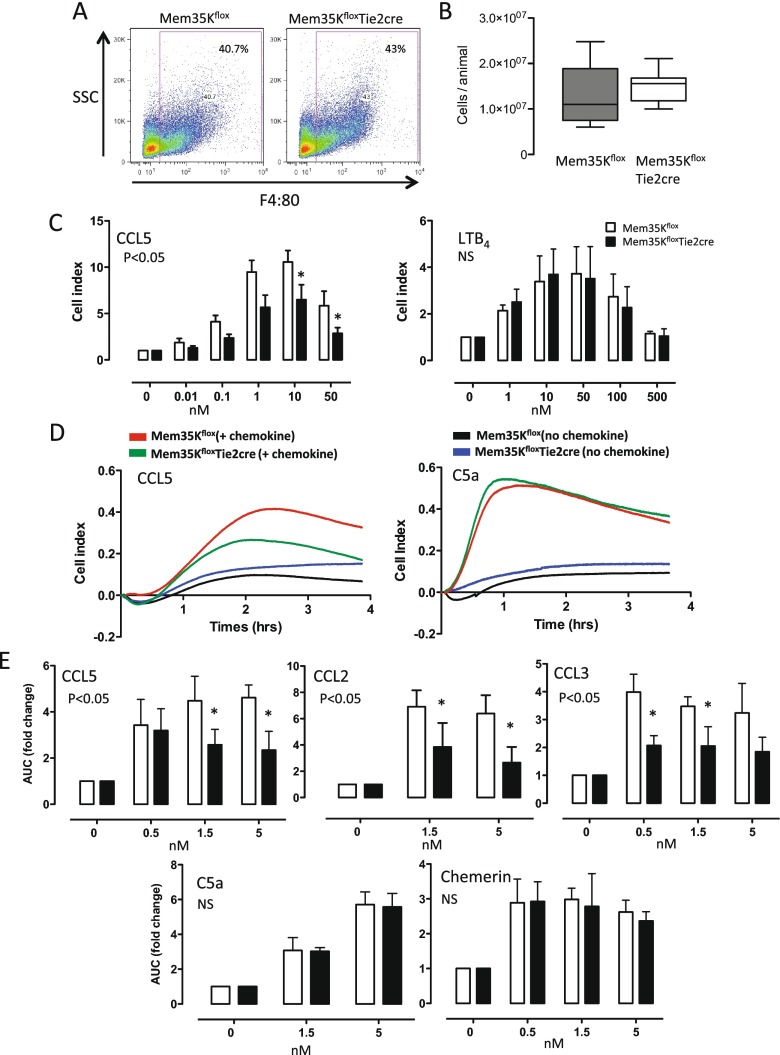
Functional expression of the Mem35K transgene. Flow cytometry using anti-F4/80 antibody and hemacytometer total peritoneal exudate cell counts indicated that the composition (**a**) and number of Biogel-elicited macrophages (**b**) was not altered in Mem35K^flox^/Tie2cre animals. **c** Modified Boyden chamber chemotaxis assays using Biogel-elicited macrophages demonstrated a significant reduction in chemotaxis to CCL5, but not to the chemoattractant LtB4. **d** Further chemotaxis studies using the xCELLigence real-time chemotaxis system demonstrated a significant inhibition of chemotaxis towards CCL5 but not towards C5a. A representative trace from the RTCA-DP software of Biogel-elicited macrophages migrating towards chemoattractant or buffer alone control is shown. **e** Quantification of the response in the xCELLigence assay showed migration towards CCL5, CCL2 and CCL3, but not towards C5a or chemerin was significantly inhibited. (*n* = 5/6 per genotype (Boyden chamber assay) or *n* = 3/4 per genotype (xCELLigence assay), two-way ANOVA, *p < 0.05)

We next tested the effects of Mem35K transgene expression on chemotaxis in primary macrophages ex vivo, first using a modified Boyden chamber assay. We first confirmed that the peritoneal recruitment of macrophages using Biogel as a eliciting agent was not altered by the expression of Mem35K. No significant difference in the number of mature F4/80 expressing macrophages recruited to the cavity was observed by flow cytometry (Fig. 3a, b). Macrophages from animals that did not express Mem35K protein demonstrated a dose-dependent chemotaxis towards both the CC-CK, CCL5 and to the non-CC-CK chemoattractant, LTB4 (Fig. 3c). Macrophages expressing Mem35K protein showed a significant reduction of the chemotactic response to the CCL5 (*P* < 0.05), whereas transgenic Mem35K expression did not affect the chemotactic response to LTB4. This inhibition of ex vivo chemotaxis is not due to alterations in CCR5 expression, which was unchanged (Fig. 2f). To further test the ability of Mem35K to specifically inhibit CC-chemokine-mediated chemotaxis, we utilised a real-time macrophage chemotaxis assay based on electrical cell impedance (ACEA Biosciences, xCELLigence). In these experiments, chemotaxis of Mem35K expressing macrophages was reduced to the CC-chemokines CCL2, CCL3 and CCL5 (*P* < 0.05), but not to the non-CC-CK chemoattractants, chemerin or C5a (Fig. 3d, e). Taken together, these data demonstrate that Mem35K expressing primary macrophages from Mem35K^flox^ Tie2cre mice show a selective inhibition of CC-CK-mediated chemotaxis.

### Functional expression of Mem35K in vivo

**Fig. 4:**
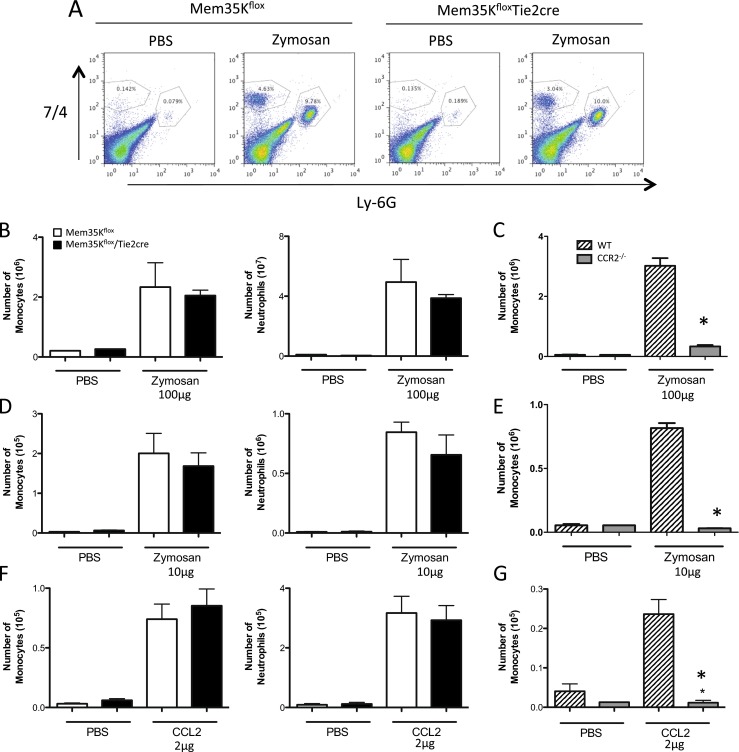
Function of the Mem35K transgene in vivo. Mice were injected ip with inflammatory stimuli, zymosan or CCL2, and recruited cells recovered at the given timepoints by peritoneal lavage. Flow cytometry was used to quantify the number of neutrophils and monocytes recruited. **a** Representative flow cytometry data from peritoneal exudates demonstrate the identification of monocytes (7/4^HI^, Ly-6G^−^) and neutrophils (7/4^HI^, Ly-6G^+^) in PBS and zymosan-treated animals (100 ug for 16 h). Mem35Kflox/Tie2cre or CCR2−/− were treated with zymosan 100 μg for 16 h (**b**, **c**) or 10 μg for 4 h (**d**, **e**) and the number of recruited monocytes and neutrophils counted. To more directly assess CC chemokine-mediated cell recruitment, mice were injected ip with 2 μg CCL2 and peritoneal lavage was performed after 4 h (**f**, **g**). See Supplementary Figure 1 for neutrophil quantification in CCR2^−/−^ mice. (*n* = 4–6 per group (Zymosan/CCL2; *n* = 1–3 saline-injected animals included per genotype to confirm an absence of pre-existing inflammation), *p < 0.05 by *T* test between genotype in zymosan/CCL2-treated animals)

Having demonstrated that transgenic expression of Mem35K by macrophages was sufficient to reduce CC-CK-mediated chemotaxis in vitro, we next tested experimental models of peritonitis to probe the ability of Mem35K to reduce chemotaxis in vivo. As chemokines have been shown to control leukocyte homeostasis, cell counts of the major circulating leukocyte populations were assessed in blood, bone marrow and spleen (Table 1) [20]. No significant alteration in the number of monocytes, neutrophils or lymphocytes was observed, confirming in vivo experiments would not be confounded by alterations in leukocyte homeostasis. To assess in vivo cell recruitment, Mem35K^flox^ Tie2cre and Mem35K^flox^ control animals were injected intraperitoneally with 100 μg zymosan. Peritoneal lavage was performed after 16 h to recover the recruited cells (Fig. 4a). The cells recruited were quantified by flow cytometry, using an absolute counting protocol. Zymosan induced the recruitment of high numbers of both monocytes and neutrophils at 16 h (Fig. 4b). However, there was no significant difference in the number of cells recruited in the Mem35K^flox^ Tie2cre or control animals. The recruitment of monocytes in this model was confirmed to be CC-CK dependent by performing a parallel experiment in CCR2−/− mice, which demonstrated a near total lack of recruitment (Fig. 4c).

**Table 1:** Leukocyte counts from blood, spleen and bone marrow in Mem35K^flox^ and Mem35K^flox^/Tie2cre animals were obtained by flow cytometry

	Mem35K^flox^	Mem35K^flox^/Tie2cre
Blood (cells × 10^6^/ml)		
Neutrophils	0.30 (±0.06)	0.44 (±0.06)
Monocytes	0.21 (±0.04)	0.25 (±0.06)
T cells	1.81 (±0.49)	2.29 (±0.59)
B cells	1.34 (±0.11)	1.30 (±0.15)
Spleen (cells × 10^6^)		
Neutrophils	0.73 (±0.08)	0.83 (±0.05)
Monocytes	0.73 (±0.22)	0.87 (±0.22)
T cells	11.9 (±2.04)	9.38 (±0.88)
B cells	17.5 (±4.56)	17.5 (±5.62)
Bone marrow (cells × 10^6^)		
Neutrophils	4.16 (±0.35)	5.15 (±0.67)
Monocytes	0.94 (±0.10)	1.44 (±0.26)

No significant effect of Mem35K expression on homeostatic cell numbers was observed. (*n* = 7/8)

Because 100 μg zymosan generates a potent sterile inflammation that takes 96 h to resolve, we next investigated a less potent model of peritonitis in order to determine whether the lack of effect of Mem35K was due to the production of inflammatory cytokines overwhelming the inhibitory capacity of cell-associated Mem35K. Mice were injected with 10 μg of zymosan for 4 h to evaluate at early leukocyte recruitment (Fig. 4d). Injection of zymosan caused recruitment of neutrophils and monocytes after 4 h in both Mem35K-expressing and non-expressing controls. However, no significant effect on the number of neutrophils or monocytes recruited was observed. Again the CC-CK dependence of this model was confirmed using CCR2−/− mice, which demonstrated a significant decrease in monocyte recruitment in this model (Fig. 4e and Supplementary Figure 1).

To directly assess whether Mem35K can inhibit specific CC-chemokines in vivo, we injected CCL2 i.p., which has previously been shown to cause monocyte recruitment to the peritoneal cavity [21]. Injection of 2 μg CCL2 caused a significant recruitment of both monocytes and neutrophils after 4 h, but no effect of Mem35K expression was seen on the recruitment of either population (Fig. 4f). Although CCL2 would be assumed to cause recruitment via the receptor CCR2, we confirmed no confounding effect of the injection procedure on monocyte recruitment and confirmed the nature of the recombinant protein injected by performing a parallel experiment in CCR2−/− mice. We demonstrated no recruitment of monocytes to CCL2 in CCR2−/− mice, confirming this model is working via CC-CK as expected; however, the migration of neutrophils was unaffected by the loss of CCR2 indicating they are migrating in response to other substances in the recombinant protein preparation (Fig. 1g and Supplementary Figure 1).

## Discussion

These studies provide new and important insights to the application of CC-CK inhibition as a cell-specific therapeutic strategy in inflammatory diseases. These new insights were gained through cell-localised expression of a broad-spectrum CC-CK inhibitor by leukocytes and endothelial cells in transgenic mice, using the flox-stop conditional expression system. Transgenic expression of the Mem35K CC-CK inhibitor protein was readily detectable and sufficient to significantly inhibit leukocyte recruitment in in vitro models of chemotaxis. However, this cell-localised expression of the CC-CK inhibitor protein did not reduce leukocyte chemotaxis in an in vivo model of inflammation. These observations suggest that cell-restricted CC-CK inhibitors will not be sufficient to limit chemotaxis of inflammatory cells in vivo and that more systemic approaches to limit CC-CK action will be required.

Previous studies with a soluble, secreted form of 35K have shown potent effects on inflammatory cell recruitment [9, 22–24]. Furthermore, high-level viral-mediated expression of Mem35K in the liver is able to reduce hepatic inflammation induced by Concavalin A [15], demonstrating that the cell-restricted expression of Mem35K can also suppress the migration of leukocytes [15]. However, whether this effect of Mem35K was achieved through competition for CC-CK binding at the surface of individual cells or through local sequestration of CC-CK in tissue could not be determined, as in vivo studies were limited to un-targeted local expression through adenoviral gene delivery, rather than through cell-targeted expression in the responder population of leukocytes. We have now achieved cell-targeted expression of Mem35K in leukocytes rather than hepatocytes that are the predominant target of adenoviral gene delivery. In transgenic leukocytes ex vivo, we again demonstrated that expression of the Mem35K is sufficient to reduce chemotaxis. In contrast, this reduced sensitivity to chemokine was not sufficient to alter leukocyte recruitment in a model of peritonitis in vivo. This finding suggests that the ability of a cell-targeted CC-CK inhibitor to exert an anti-inflammatory effect in vivo is more reliant on the ability of the molecule to sequester chemokines in the tissue, rather than by exerting a selective and cell-specific inhibition of chemokine receptor signalling at the surface of individual leukocytes. Such a cell-selective inhibition of chemokine signalling by Mem35K may require higher expression—it is of note that the migration of Mem35K-transfected cells towards CC-CK is almost completely ablated in our previous study, but is only blunted in our transgenic leukocytes [15]. Alternatively, cells expressing Mem35K in vitro may show a more potent inhibition of CC-CK as they are ‘produced’ in a system that lacks CC-CK, therefore enabling the full complement of Mem35K to bind exogenously applied CC-CK, in contrast to cells in vivo, where cumulative saturation of Mem35K by CC-CK may ultimately diminish any functional impact on inflammatory cell recruitment.

Analysis of the structure of 35K bound to CCL4 reveals that 35K binds the residues responsible for CC-CK receptor binding. As a result, chemokines bound to 35K are unable to activate downstream signalling. We have previously shown that soluble 35K protein present in plasma binds chemokine and renders it inactive by demonstration that whilst chemokine levels in plasma are not decreased, or even are increased, in 35K expressing animals, that the chemokine mediated biological activity of those fluids is reduced [9, 24].

Clear evidence that the expression of a competitor for CC-CK binding is sufficient to regulate inflammation is provided by the D6 decoy chemokine receptor that regulates the resolution of inflammation by scavenging chemokines. Whilst D6^−/−^ mice do not show any obvious phenotype in the absence of an inflammatory challenge, these mice show a significant increase in concentration of its ligands in plasma and inflamed tissue and the development of a more severe inflammatory response [25, 26]. This action of D6 demonstrates that expression of a cell-surface CC-CK binding molecule is a mechanism by which inflammation can be regulated in vivo. Given this is the case, then it may follow that Mem35K, in addition to the level of expression that can be achieved by a single copy transgene, may lack other features of its regulation that are required to act in this fashion. D6 undergoes rapid recycling between the plasma membrane to the endosome; however, in contrast to chemokine receptors that also internalise following ligand binding, D6 surface expression is not downregulated by prolonged exposure to its ligand [27]. Indeed, there is little evidence that D6 activity is regulated by expression, rather that changes in the rate of cell surface recycling allows increased activity during inflammation [28]. Mem35K lacks an intracellular signalling domain, so is unable to undergo the controlled endocytic process used by D6. Instead, membrane expression can only be controlled by gene expression, using a CMV-driven promoter that is not highly regulated by inflammation [29]. The successful expression by adenovirus of this protein may be in part due to the high level of expression that is present acutely following infection. The fate of CC-CK once bound to 35K or Mem35K is unknown, so whether the binding capacity of each individual molecule is rapidly exhausted is key to understanding the utility of this molecule in therapy. If each molecule can only bind a single CC-CK molecule (as suggested by the co-crystal structure with CCL4), then a key aspect of this therapy is in sustaining a continuous high level of ‘free’ Mem35K. The binding capacity of Mem35K is not exhausted during the development and circulation of the transgenic leukocytes as the primary Biogel-elicited cells still demonstrate an in vitro phenotype. The Mem35Kflox/Tie2cre mouse also exhibits expression in endothelial cells, and despite this additional expression, which might be expected to exert a bystander effect on active chemokine availability, no in vivo reduction of inflammation is seen. Although we have not probed the functionality of the endothelial cell-expressed Mem35K in detail, we would hypothesise that the limitations of the level and non-regulated manner of expression would apply as they do to the leukocyte-expressed molecule.

Therapies that act by competition can be an effective strategy, since anti-TNF targets the soluble component to compete with TNF action at a systemic level [30]. However, it is likely that cell-targeted therapies must be potent and regulated by inflammation, if they are a competitive inhibitor, or block the cell-autonomous part of the pathway such as the receptor. Furthermore, such a strategy is likely to require a sustained, high level of unbound inhibitor in order to exert a functional effect (as exemplified by viral delivery systems), but as a purified protein may be expensive and require frequent dosing.

In conclusion, expression of the cell-associated CC-CK inhibitor, Mem35K, by leukocytes effectively inhibits CC-CK action in vitro, but this cell-associated inhibition of CC-CK is not effective in blockade of leukocyte recruitment in vivo. Strategies to reduce inflammation by CC-CK binding will require high-level CC-CK binding to reduce CC-CK action in the whole tissue or systemically, making cell-targeted CC-CK inhibition strategies less likely to be effective as a therapeutic strategy.

## Electronic supplementary material

Below is the link to the electronic supplementary material.

ESM 1 (PDF 651 KB)
