# Supplementary material for: Contrasting in vitro vs. in vivo effects of a cell membrane-specific CC-chemokine binding protein on macrophage chemotaxis
Source: J Mol Med (Berl). 2014 Aug 1;92(11):1169–78. doi: 10.1007/s00109-014-1194-6 (PMC4206339; doi:10.1007/s00109-014-1194-6)
Supplement: Supplementary file 1 — (PDF 651 KB) [file 109_2014_1194_MOESM1_ESM.pdf]

Supplementary Materials and Methods

Contrasting in vitro vs. in vivo effects of a cell membrane-specific CC-chemokine binding protein on macrophage chemotaxis

Eileen McNeill[1,2], Asif J Iqbal[3], Jyoti Patel[1,2], Gemma E White[3], Daniel Regan-Komito[3], David R Greaves[3], Keith M. Channon[1,2]

[1]*Division of Cardiovascular Medicine, University of Oxford*

[2]*Wellcome Trust Centre for Human Genetics, University of Oxford*

[3]*Sir William Dunn School of Pathology, University of Oxford*

Corresponding author: *Prof. Keith Channon, Division of Cardiovascular Medicine, University of Oxford, John Radcliffe Hospital, Oxford OX3 9DU, U.K.*

Email: keith.channon@cardiov.ox.ac.uk Tel: +44 1865 572783

Animals

All animal studies were conducted with ethical approval from the Local Ethical Review Committee and in accordance with the UK Home Office regulations (Guidance on the Operation of Animals, Scientific Procedures Act, 1986). Homozygous CCR2-/- (Jax strain B6.129S4-Ccr2tm1Ifc/J) mice were bred in-house and control C57bl/6J mice were purchased from Harlan UK.

Cell transfection studies

HEK 293 cells were transfected with the Mem35K plasmid or empty plasmid control using GeneJuice (Merck Millipore) and harvested after 24 hours for western blotting, confocal microscopy and flow cytometry. Cell surface Mem35K expression was confirmed by detection of the HA-tag using an anti-HA antibody (Miltenyi).

Blotting and Immunoprecipitation

The Mem35K protein was detected by standard western blotting using mouse monoclonal anti-HA (Roche), goat polyclonal anti-35K (R&D systems) or mouse monoclonal anti-GAPDH (Chemicon) antibodies.

To detect mem35K in primary cells and tissues lysates Mem35K protein was immunoprecipitated using anti-HA agarose beads (Sigma Aldrich). Cell lysates were produced using Cell-lytic MT (Sigma) lysis buffer with protease inhibitors (Roche) by homogenization (tissues) or freeze/thaw (cells). The resulting lysate was centrifuged to remove insoluble material and protein concentration was measured using a colourimetric protein assay (BCA, Pierce). The lysates were all adjusted to 1mg/ml using the Cell-lytic MT lysis buffer and 50ul of anti-HA The resulting protein solution was subjected to western blotting as above.

Breeding Mem35K transgenic and Tie2cre transgenic mice

The Mem35K transgene is inserted into the X chromosome, therefore female heterozygous mice manifest mosaic expression, due to random X inactivation. Due to this phenomenon only hemizygous male and homozygous female Mem35K mice are used for experiments. No gene dosage effect is present as both male and female mice will only express Mem35K from one allele. Initial characterization was carried out on C57Bl/6J backcross generation 3 animals, with key findings such as peritonitis and chemotaxis data reproduced on backcross 6 animals. The Tie2cre transgene is active in the female germline, as such only male animals are used to establish breeding pairs to maintain conditional expression. Mice were genotyped using primers targeted against the wildtype HPRT allele, floxed sequence and in a separate reaction for the presence of the cre sequence.

HPRT

5’ CATCGCATAAACTGGGTGTG 3’

5’ TGAATCCCAAGCCATCTAGG 3’

Mem35K

5’ ACTTCAAGATCCGCCACAAC 3’

5’ AGTTCTGCCAGCTCCTTCTG 3’

Cre

5' GCATAACCAGTGAAACAGCATTGCTG 3'

5' GGACATGTTCAGGGATCGCCAGGCG 3'

Genomic DNA production and excision PCR

Genomic DNA for detection of the excised allele was produced using the Qiamp kit (Qiagen).The floxed and excised allele were detected using the following primers:

5'-TGCAGATGAACTTCAGGGTCAGCTTGC-3

5'-AAATCTGTGCGGAGCCGAAATCTGG-3'

5’-GGTGGAAACAACGGTGGCAGCA-3’

The floxed allele yields a 536bp product and the excised allele a product of 876bp.

Primary cell preparations:

Bio-Gel Elicitation of Primary Mouse Macrophages.

Mice were injected intraperitoneally with 1 ml of 2% Bio-Gel P-100 fine polyacrylamide beads (Biorad) suspended in PBS. Mice were sacrificed after 4 days and the peritoneum lavaged with 10 ml of ice-cold PBS/5 mM EDTA. The cellular composition of the recruited cells was confirmed with by flow cytometry using anti-f4:80 antibody (Adb Serotech).

Primary endothelial cell isolation

Primary endothelial cells were isolated from PBS perfused lung tissue. Lungs were finely minced and digested in DMEM containing 0.18U/ml Liberase Blendyme 3 (Roche) and 0.1mg/ml DNase I (Roche) for 60 minutes at 37°C. Positive selection for endothelial cells was achieved using anti-CD31 (BD Biosciences) conjugated sheep anti-Rat IgG Dynabeads. The selected cells were cultured for 5 days in gelatin coated 6 well plates in EBM endothelial cell media prior seeding onto gelatin coated coverslips overnight.

Blood, Bone Marrow and Spleen Leukocytes

Single cell suspensions of splenocytes and bone marrow cells were obtained using standard protocols (1). Blood samples were taken directly into an EDTA coated tube (Teklab). All cell populations were stained with monoclonal antibodies directed against CD3, B220, Ly-6G (all BD Biosciences) and the 7/4 antigen (AbD Serotech). The total cell population was gated by forward scatter and side scatter then interrogated for expression of the relevant cells markers compared to isotype controls (T-cells – CD3^{+}, B-

Cells – B220$^+$, Neutrophils – 7/4^{HI}, Ly-6G$^+$ or Monocytes – 7/4^{HI}, Ly-6G$^-$). Cells were enumerated by an absolute count as described below.

Flow cytometry

All flow cytometry was performed using a DAKO CyAn cytometer and Summit software (both Beckton Coulter). Data was analysed using Flow Jo software (TreeStar Inc). Where cells were enumerated an absolute count protocol was used where cells were quantifed by ratio to a known number of fluorescent beads spiked into the sample prior to analysis ((2)). For quantification of fluorescence the geometric mean fluorescence of the histogram population was used.

Immunofluorescence and confocal microscopy

Tissuetek OCT embedded fresh frozen aortic sections,HEK 293 cells, primary endothelial cells or macrophages were mounted on coverslips and fixed (4% Paraformaldehyde), permeabilised (0.1% Triton X-100) and blocked (PBS/10%FBS) prior to incubation with anti-GFP (Invitrogen) or anti-CD31 (BD biosciences). Secondary antibodies (anti-rabbit Alexa 488 and anti-rat Alexa 568) were applied followed by counterstaining with DAPI (Calbiochem) and mounting in Mowiol (Calbiochem).

All confocal microscopy was performed using a Zeiss confocal with a 63x oil immersion objective and LSM software with multi-track acquisition setup.

293 cell CCR5-mediated chemotaxis

CCR5 mediated chemotaxis was assessed using 293 cells transfected with CCR5, EGFP and mem35K (or empty plasmid control) in Chemotx plates (Neuroprobe) as described previously (3). Each experimental sample was analyzed in triplicate, and three separate images were quantified for each membrane.

CCL5 binding assay and CCR5 expression.

Binding of human CCL5 to Biogel-elicited macrophages was assessed using a Fluorokine CCL5 assay, according to the manufacturer's instructions (R&D Systems). Macrophages

were identified as FSC^{HI}/SSC^{HI} cells. Parallel cell isolations were used to assess cell surface CCR5 expression by flow cytometry using an anti-CCR5 antibody (BD Biosciences). CCL5 binding, CCR5 expression and endogenous GFP fluorescence was assessed in n=3-6 animals per genotype and significant alterations in any parameter were assessed by T-test, with $p<0.05$ being judged statistically significant.

Chemotaxis – Boyden Chamber Assay

4×10^5 Bio-Gel-elicited cells were harvested and placed on 96-well Neuroprobe membranes (6-mm diameter, 8-μm pore size; ChemoTX) in RPMI supplemented with 25 mM Hepes (Life Technologies) and 0.1% BSA (Sigma-Aldrich). The cells were allowed to migrate toward CCL5 (PeproTech) or LtB4 (Sigma), or serum-free medium for 4 hours. After 4hours the cells remaining on top of the membrane were removed and the upper membrane wiped clear of cells. Migrated cells on the underside of membrane were fixed (4% paraformaldehyde) and stained with DAPI. Two photos per membrane and a minimum of three replicate wells per treatment were imaged using a fluorescence microscope. The number of cell having migrated was calculated using an automated nuclei count function in Image Pro Plus software (Media Cybernetics). Migration indices were obtained by dividing the number of cells recruited to treatments by the number migrating to serum-free medium.

xCELLigence real-time Chemotaxis assay

Biogel elicited cells were subjected to chemotaxis assays in CIM-16 well plates using an xCELLigence RTCA-DP instrument as described previously ((4)). Briefly, agonists were loaded into the lower chamber of the CIM-16 plate and the upper chamber attached. The electrode embedded membrane was equilibrated with prewarmed buffer for 30 mins prior to addition of $8x10^6$ Biogel-elicited cells. Migration was assessed every 5s for 4 hours in total. Data were normalized to wells that received media alone and analysis of area under the curve was carried out with RCTA Software version 1.2.1.

Peritonitis models

Zymosan-induced peritonitis was performed as described previously ((5)). In brief, mice were injected intraperitoneally with 10 μg or 100 μg of zymosan A (Sigma-Aldrich) or 2ug JE (Peprotech EC) diluted in 0.5 ml of PBS or PBS alone. Four hours (JE and 10μg zymosan) or 16 hours (100μg zymosan) later mice were sacrificed and the peritoneal cavity was lavaged with 5 ml of PBS/2 mM EDTA. Peritoneal exudate cells were stained with antibodies against Ly6G (BD Biosciences,) and 7/4 (AbD Serotec) and analyzed by flow cytometry to assess neutrophil and monocyte recruitment.

Statistics

All statistical analyses were performed using Prism software version 5 (Graph Pad). All values are expressed as the mean ± SEM. Data were analyzed using a two-way analysis of variance and the Bonferroni *post hoc* test of significance was used to test between treatment groups at individual dose or time points if appropriate. Where a single dose or timepoint was analysed a one-way ANOVA was used. A value of $P<0.05$ was the criterion of significance.

References

1. Hobbs, J. A., May, R., Tanousis, K., McNeill, E., Mathies, M., Gebhardt, C., Henderson, R., Robinson, M. J., and Hogg, N. (2003) Myeloid cell function in MRP-14 (S100A9) null mice. *Mol Cell Biol* **23**, 2564-2576
2. Hubl, W., Iturraspe, J., Martinez, G. A., Hutcheson, C. E., Roberts, C. G., Fisk, D. D., Sugrue, M. W., Wingard, J. R., and Braylan, R. C. (1998) Measurement of absolute concentration and viability of CD34+ cells in cord blood and cord blood products using fluorescent beads and cyanine nucleic acid dyes. *Cytometry* **34**, 121-127
3. Bursill, C. A., McNeill, E., Wang, L., Hibbitt, O. C., Wade-Martins, R., Paterson, D. J., Greaves, D. R., and Channon, K. M. (2009) Lentiviral gene transfer to reduce atherosclerosis progression by long-term CC-chemokine inhibition. *Gene Ther* **16**, 93-102
4. Iqbal, A. J., Regan-Komito, D., Christou, I., White, G. E., McNeill, E., Kenyon, A., Taylor, L., Kapellos, T. S., Fisher, E. A., Channon, K. M., and Greaves, D. R. (2013) A real time chemotaxis assay unveils unique migratory profiles amongst different primary murine macrophages. *PLoS One* **8**, e58744

5. Cash, J. L., White, G. E., and Greaves, D. R. (2009) Chapter 17. Zymosan-induced peritonitis as a simple experimental system for the study of inflammation. *Methods in enzymology* **461**, 379-396

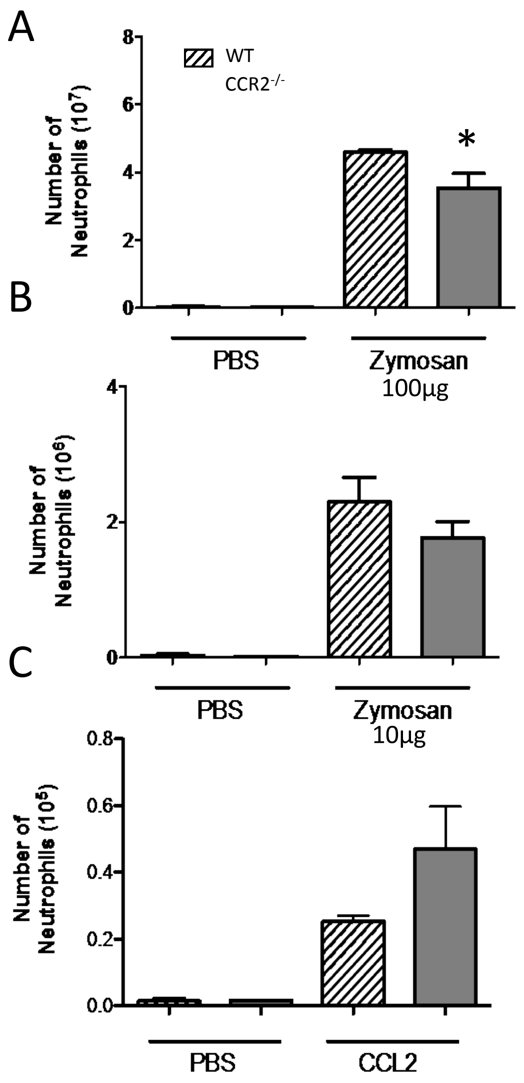

Supplementary Figure 1: Function of the Mem35K transgene *in vivo*. (A) CCR2-/- were treated with zymosan 100μg for 16 hours or 10μg for 4 hours (B) and the number of recruited neutrophils counted. (C) To more directly assess CC chemokine mediated cell recruitment mice were injected ip with 2μg CCL2 and peritoneal lavage was performed after 4 hours and neutrophils counted. (n=3-6 per group, * $p<0.05$ by T-test). n=1-2 saline injected animals included to confirm an absence of pre-existing inflammation. Lack of CCR2 caused a small but significant decrease in neutrophil recruitment to 100ug at 16 hours after injection, but not to any other inflammatory stimulus injected. The neutrophil response to CCL2 injection was not ablated in the absence of CCR2 indicating neutrophil recruitment is likely a result of contaminants in the CCL2 preparation.
